# Clustering and Tracking the Stability of Biological CVD Risk Factors in Adolescents: The Malaysian Health and Adolescents Longitudinal Research Team Study (MyHeARTs)

**DOI:** 10.3389/fpubh.2020.00069

**Published:** 2020-03-17

**Authors:** Nithiah Thangiah, Karuthan Chinna, Tin Tin Su, Muhammad Yazid Jalaludin, Nabilla Al-Sadat, Hazreen Abdul Majid

**Affiliations:** ^1^Department of Social and Preventive Medicine, Faculty of Medicine, Centre for Population Health (CePH), University of Malaya, Kuala Lumpur, Malaysia; ^2^Faculty of Health and Medical Sciences, School of Medicine, Taylor's University, Selangor, Malaysia; ^3^Jeffery Cheah School of Medicine and Health Sciences, Monash University, Selangor, Malaysia; ^4^Department of Paediatrics, Faculty of Medicine, University of Malaya, Kuala Lumpur, Malaysia; ^5^T.H. Chan School of Public Health, Harvard University, Boston, MA, United States; ^6^Department of Nutrition, Faculty of Public Health, Airlangga University, Surabaya, Indonesia

**Keywords:** clustering, tracking, risk factors, cardiovascular diseases (CVD), adolescents

## Abstract

**Background:** Cardiovascular disease (CVD) risk factors tend to cluster and progress from adolescence to young adulthood. Reliable and meaningful clustering of CVD risk factors is essential to circumvent loss of information. Tracking adverse and high-risk profiles of adolescents is hoped to curb CVD progression later in life. The study aims to investigate the clustering of biological CVD risk factor among adolescents in Malaysia and the transitions between clusters over time.

**Method:** The Malaysian Health and Adolescents Longitudinal Research Team study (MyHeARTs) examined school students aged 13 in 2012 and re-examined them in 2014 and 2016. In a two-stage stratified cluster sampling, 1,361 students were recruited, of which, 1,320 had complete data. In the follow-up, there were 881 and 637 students in 2014 and in 2016, respectively. Pearson's correlation coefficients were used to identify and remove highly correlated CVD risk factors. All risk factors were standardized into z-scores. The hierarchical and non-hierarchical (k-means) cluster analyses were used to classify students into high, medium and low risk clusters in each screening year. The tracking and stability of cluster transitions through cross-classification were enumerated with Pearson's inter-age correlations and percentages.

**Results:** Three significant clusters of high, medium and low risk groups were derived from the clustering of eight biological CVD risk factors. The transitions between risk clusters from one screening year to the other were categorized as either stagnant, improved or adverse. The number of students who had adverse transitions increased from 15.5% (13–15 year) to 19.5% (15–17 year), 13.8 to 18.2% among the girls and 19.9 to 22.8% among the boys. For girls, the number of them who remained at high risk over the two transition periods were about the same (13.6 vs. 13.8%) whereas for boys, the percentage reduced from 14.6 to 12.3%.

**Conclusion:** Over time, more than 12% of adolescents remained in the high risk cluster. There were sizable adverse transitions over time as more adolescents appear to be shifting toward an increased risk of having CVD. Collaborative and constant measures should be taken by parents, school, health promotion boards and policy makers to curb the multiplicative effect of clustering CVD risk factors among adolescents.

## Introduction

Non-communicable diseases (NCDs) contribute to 71% of deaths globally. Cardiovascular diseases (CVD) is one of the primary NCDs (1). In 2016, WHO reported that 17.9 million or 44% of NCD deaths were due to CVD. Three quarter of these deaths occurred in low and middle income countries. In Malaysia, CVD is the leading cause of death as it accounted for an estimated 35% of all deaths due to NCDs. The common risk factors for CVD, namely heart attack and stroke, are hypertension, hypercholesteremia, diabetes and overweight/obesity. The 2015 National Health and Morbidity Survey (NHMS) reported 63% of adults had at least one of these risk factors and that children and adolescents are as vulnerable to these risk factors (2). Among ASEAN countries, Malaysia is ranked second highest in terms of childhood obesity, with a 12.7% prevalence of age standardized overweight (1).

It is widely known that biological risk factors for cardiovascular diseases (CVD) such as blood pressure, body composition, body lipid, body fitness and blood glucose tend to cluster (3–5). The clustering of these risk factors has a multiplicative effect that induces an elevated risk of CVD (6–9). These risk factors start early in life during adolescence and persist into adulthood (10). In adolescence, clustering of multiple risk factors leads to initial stages of atherosclerosis. Streaks of fat, cholesterol and fibrous plaques begin to accumulate in the artery walls at a very young age of 10 and slowly accumulates over time (6, 11, 12). These atherosclerotic lesions are usually not manifested until the child becomes an adult. As such, tracking the clustering of multiple risk factors of CVD from young adolescence is of vital importance (13, 14). Tracking is often done by evaluating the transitions in risk clusters over time. Clusters are said to be stable when the transitions between risk clusters remain the same.

Although several risk clustering and tracking methods exist, most findings show high risk clusters tend to be moderately to highly stable (13, 14). A study among schoolchildren, aged 15 to 19 years, in Denmark measured total risk scores as the sum of seven risk factors; systolic and diastolic blood pressure, total cholesterol, HDL-cholesterol, triglyceride, smoking and skinfold thickness. The tracking of total risk score over 8 years found a significantly high number of subjects remaining in upper quintiles (*r* = 0.85) (15). Similarly, another study from the same cohort of Danish adolescents reported the probability of being in the upper quartile of two or more risk factors (TC:HDL ratio, triglyceride, systolic blood pressure, and body fat) at the first examination was six times greater than the second examination 8 years later (16). A study of young Finnish children aged 6–18 years, tracked the transitions of high-risk groups using 3 risk factors; total cholesterol, HDL-cholesterol and diastolic blood pressure. The 6-years tracking of risk factors found about 25% of children remained in high risk groups with extreme tertiles (17). The Bogalusa Heart Study examined the persistence of clustering multiple CVD risk factors consisting adverse levels of systolic blood pressure, TC:HDL ratio and plasma insulin. The 8 years follow up study of individuals aged 5–17 years old showed that 61% of the individuals in the highest quintile of the multiple index score maintained their rankings (18). The Aerobics Center Longitudinal Study measured a composite risk factor score of waist circumference, HDL-cholesterol, triglyceride, glucose and mean arterial blood pressure and found them to track moderately well (*r* = 0.56) from a mean age of 15.8–26.6 years (19).

The purpose of the study was to investigate the clustering of biological CVD risk factors and its' transitions over time, among adolescents in Malaysia. The first part of the study examined if the relevant CVD risk factors identified among the adolescents clustered in a reliable and meaningful way. In the second part of the study, the transitions from year 2012–2014 and 2014–2016 were examined separately in order to track the stable (especially those who remained in high risk clusters), adverse and improved changes in clusters among the adolescents. We hypothesized adolescents in high risk clusters can be clearly distinguished to indicate high risk of behavioral patterns.

## Methods

The STROBE statement was adhered in reporting this study.

### Data Source

The Malaysian Health and Adolescents Longitudinal Research Team study (MyHeARTs) is an inaugural initiative conducted in Malaysia. The study was designed to examine the trends of risk factors of non-communicable diseases among an adolescent cohort. The respondents were followed from the ages of 13–17. Measurements were taken at baseline (13 years), 15 years and 17 years.

The formula used to calculate sample size was *n* = $\left(\frac{z^{2}\;\times p\;\times q}{r\;\times \;e^{2}}\right)\times design\;effect$ where *z* = standard normal deviate set at 1.96 at 5% level for two tailed test, *p* = estimated prevalence of adolescents aged 13–15 who smoked in school at 33%, *q* = 1-p, *r* = response rate and *e* = precision level. A total sample of 1,500 students were estimated. A two-stage stratified cluster sampling design was used in this study. In the first stage, 15 schools (eight urban-based and seven rural-based) were randomly selected based on the calculated sample size (20). The selection was based on a complete list of secondary public schools (sampling frame) located in the Federal Territory of Kuala Lumpur (capital city of Malaysia) and the central and northern zone of Peninsular Malaysia, specifically in the states of Selangor and Perak. In the second stage all 13-years-old students from the selected schools were invited to enroll in the study. There were 1,361 students at baseline in 2012 and subsequent follow ups were conducted in 2014 and 2016.

Data collection and examination of students were carried out during school hours with approvals from respective school administration and Ministry of Education. Self-administered questionnaires were distributed among students. Trained enumerators present during the data collection period ensured the smooth flow. Information on socio-demographics, lifestyle, health background and high risk behaviors were collected. More details about the study procedure and sampling have been reported elsewhere (20). All procedures involving human subjects were approved by the Ethics Committee of University Malaya Medical Centre (Ref. No. 896.34).

### Response Rate and Follow-Up

Of those invited, 2,694 students agreed to participate with written informed consent from their parents. Out of those 2,694 students, only 1,361 of them participated in the study (51%). In the follow-ups there were 925 and 654 students in 2014 and 2016, respectively. The attrition rate was 32.0% in the first follow up and 29.3% in the second follow. These attrition rates are very common in cohort studies (21) and was mainly due to withdrawal of consent and shifting to different schools. In 2012, out of the 1,361 students, 1,320 students had complete measurements for all the risk factors considered in this study. Out of the 1,320 students, 881 and 637 students had complete measurements for the required risk factors in 2014 and 2016, respectively.

### Measurements

Eleven CVD risk factors were considered in this study; systolic blood pressure, diastolic blood pressure, total cholesterol: HDL ratio, HDL cholesterol, LDL cholesterol, triglyceride, total cholesterol, body fat, waist circumference, BMI, and blood glucose. Details of each measurement are described briefly as they have been presented elsewhere (20). Risk factors that were considered for analysis is explained in variable selection.

#### Blood Pressure

Both systolic and diastolic blood pressure of the students were measured by medical doctors. Measurements were taken three times repeatedly with a 2-min interval between each reading. The average reading was calculated. At each measurement, the student was seated with right upper arm positioned at the level of the heart with both feet flat on the floor. The measurements were taken using a stethoscope and a mercurial sphygmomanometer (CK-101C, Spirit Medical Co., Taiwan).

#### Body Lipid

A total of 15 ml of fasting blood was withdrawn from each student by a phlebotomist. The students were asked to fast for at least 10 h prior to blood taking. All blood samples were sent to the hospital laboratory before storing it temporarily at 4°C in a cool box upon blood withdrawal. The blood samples were processed at the field laboratories in each state. The samples were spun and stored as serum and divided into several aliquots of 0.5 ml of serum for individual tests. In a plain test tube, 3 ml of blood was collected for the measurement of fasting lipids (Advia Chemistry, Siemens, Germany—triglyceride, total cholesterol, high density lipoprotein cholesterol and low-density lipoprotein cholesterol).

#### Body Composition

Waist circumference (WC) was measured using a circumference measuring tape (Seca 201, Seca, UK). The WC was measured at the midpoint between the lowest rib margin and the iliac crest and recorded to the nearest 0.1 cm. The percentage of body fat was measured using the Tanita portable Body Composition Analyzer SC-240 MA (22). The machine was placed on a flat surface and each student was asked to step on the platform, bare-footed. The percentage of body fat was recorded to the nearest decimal.

## Statistical Analysis

### Variable Selection

All analyses were performed using IBM SPSS Statistics (version 22; SPSS Inc., Chicago, IL, USA). The first part of the study involved clustering the selected CVD risk factors. Preliminary analyses on 11 initially selected risk factors (systolic blood pressure, diastolic blood pressure, total cholesterol: HDL ratio, HDL cholesterol, LDL cholesterol, triglyceride, total cholesterol, body fat, waist circumference, BMI, and blood glucose) was done prior to clustering. First, for each risk factor, extreme values (more than three standard deviations above or below mean) were removed as it may result in too few observations in any one cluster (23, 24). Next, correlation coefficients between the 11 traditional CVD risk factors were examined. Variable reduction was done by looking at high correlation values between variables. CVD risk factors with correlations more than 0.9 were excluded from the clustering analysis to avoid over-representation of any single factor (25, 26). Finally, the remaining risk factors were transformed into z-scores due to varying means and variances (27). Standardizing the risk factors into z-scores was essential to identify subjects with similar characteristics so that clusters of homogeneous risk factors can be segmented.

### Cluster Analysis

A two stage cluster analysis combining hierarchical and non-hierarchical (k-means) clustering methods were used (23, 28, 29). At the first stage, hierarchical method based on squared Euclidean distance and Ward's minimum variance algorithm was applied to form initial cluster centers. These non-random starting points are then applied at the second stage of k-means clustering to identify homogeneous subgroups (clusters) of students at high, medium or low risk of CVD. The reliability of the cluster solution was examined by splitting the sample into two random subsamples (23, 30). The clustering procedure was repeated to check for agreement (Kappa, κ) in cluster solution between subsamples and total sample (31). Lastly, the resulting clusters were profiled based on sociodemographic and CVD risk factors using descriptive cross-tabulation (chi-square). Since the risk factors deviated from the normal distribution, results were presented using median (lower quartile, upper quartile) and were compared using the non-parametric Kruskal Wallis (*post-hoc* Dunnett T3).

### Tracking/Stability

In the second part of the analysis, all steps above were repeated for each follow up year from 2012 to 2014 and 2016. Each year, students were clustered cross-sectionally, into low, medium and high risk clusters based on the eight finalized risk factors. A 3 × 3 matrix was constructed to show the number of students belonging to the corresponding clusters in two subsequent years. Subjects are considered to track well if the transitions between two evaluation periods maintained their ranks over time (32). Nine possible transitions between low, medium and high risk clusters were examined.

The transitions from year 2012 to 2014 and from 2014 to 2016 was examined separately to further track the transition of students over time. For this part of the analysis, the nine identified transitions were broadly categorized into five transitions of stable; moving from low to low, medium to medium, high to high, adverse (low to high, low to medium and medium to high) and improved (medium to low, high to medium and high to low). Subsequently, the percentage change of each CVD risk was calculated for the periods from 2012–2014 as $\left(\frac{x_{2014-}x_{2012}}{x_{2012}}\times 100\right)$ and from 2014–2016 as $\left(\frac{x_{2016-}x_{2014}}{x_{2014}}\times 100\right)$. The differences in the percentage change of each CVD risk factor among the 5 identified transitions were compared using the non-parametric Kruskal Wallis test.

## Results

**Figure 1:**
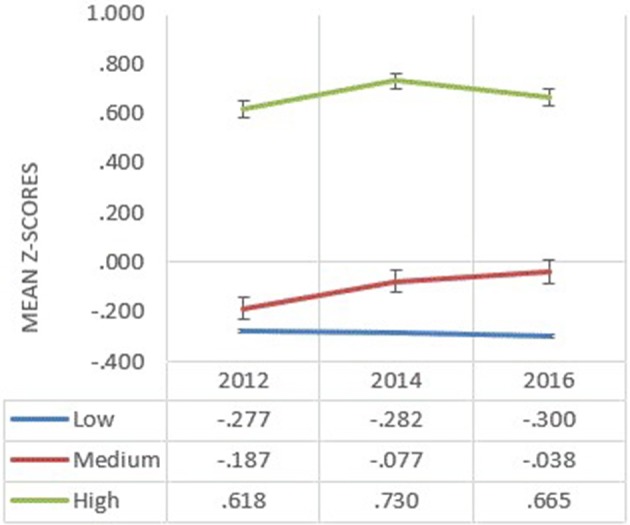
Mean of final cluster centers of all the biological risk factors in z-scores by each risk cluster (low, medium, and high) and year in 2012, 2014, and 2016.

CVD risk factors with correlations more than 0.9 were excluded from the clustering analysis to avoid over-representation of any single factor (25, 26). Based on this, the total number of risk factors considered dropped from 11 to 8; systolic blood pressure, diastolic blood pressure, TC:HDL ratio, HDL cholesterol, LDL cholesterol, triglyceride, body fat and waist circumference. Cluster analysis based on eight CVD risk factors of the adolescents resulted in them being in either one of the three distinct cluster solutions of low, medium or high risk of CVD in each year, cross-sectionally. Each cluster was distinguished based on the mean of final cluster centers in *z*-values, as shown in Figure 1. Subjects from the high risk cluster in all 3 years have distinctively higher means in final cluster center as compared to the subjects from the medium and low risk clusters. The reliability of the cluster solutions was determined by randomly splitting the total sample into two subsamples. The degree of agreement between the new clusters obtained from random subsample and those of the total sample are calculated. In 2012 and 2014, the agreement was excellent with κ = 0.962 (ρ < 0.001) and κ = 0.959 (ρ < 0.001), respectively. In 2016, the kappa statistic was 0.64 (ρ < 0.001) indicating a fairly high degree of replication as well.

Sociodemographic characteristics of the clusters by each year are presented in Table 1. The associations between cluster groups and gender, ethnicity, locality, origins by states, gross household income and highest education of parent were tested using chi-square tests. In 2012, only gender, ethnicity and states were found to be significantly associated with the clusters. In 2014, only gender and gross household income were significantly associated with cluster membership. In 2016, all relationships were significant except for ethnicity, gross household income and highest education of parent. The association between gender and cluster membership was statistically significant at all three time periods, with more females in each risk-cluster group, χ^2^(2) = 9.87, ρ < 0.01 *in* 2012; χ^2^(2) = 8.46, ρ < 0.05 *in* 2014; χ^2^(2) = 37.36, ρ < 0.001 *in* 2016.

**Table 1:** Sociodemographic characteristics of adolescents by clusters and by year.

	2012					2014					2016				
	Low *n* = 446	Medium *n* = 529	High *n* = 345	Total *n* = 1320	χ^2^	Low *n* = 346	Medium *n* = 365	High *n* = 170	Total *n* = 881	χ^2^	Low *n* = 289	Medium *n* = 201	High *n* = 147	Total *n* = 637	χ^2^
	*N* %	*N* %	*N* %	*N* %		*N* %	*N* %	*N* %	*N* %		*N* %	*N* %	*N* %	*N* %	
**Gender**															
Male	146	223	140	509	9.866^**^	99	140	64	303	8.462^*^	55	89	39	183	37.358^***^
	32.7	42.2	40.6	38.6		28.6	38.4	37.6	34.4		19.0	44.3	26.5	28.7	
Female	300	306	205	811		247	225	106	578		234	112	108	454	
	67.3	57.8	59.4	61.4		71.4	61.6	62.4	65.6		81.0	55.7	73.5	71.3	
**Ethnic**															
Malay	347	455	275	1077	36.004^***^	272	286	134	692	10.308	223	160	108	491	6.126
	77.8	86.0	79.7	81.6		78.6	78.4	78.8	78.5		77.2	79.6	73.5	77.1	
Chinese	24	45	30	99		19	33	14	66		23	15	17	55	
	5.4	8.5	8.7	7.5		5.5	9.0	8.2	7.5		8.0	7.5	11.6	8.6	
Indian	52	20	31	103		37	24	18	79		32	15	12	59	
	11.7	3.8	9.0	7.8		10.7	6.6	10.6	9.0		11.1	7.5	8.2	9.3	
Others	23	9	9	41		18	22	4	44		11	11	10	32	
	5.2	1.7	2.6	3.1		5.2	6.0	2.4	5.0		3.8	5.5	6.8	5.0	
**Locality**															
Urban	225	279	191	695	1.887	196	202	90	488	0.634	173	90	78	341	10.863^**^
	50.4	52.7	55.4	52.7		56.6	55.3	52.9	55.4		59.9	44.8	53.1	53.5	
Rural	221	250	154	625		150	163	80	393		116	111	69	296	
	49.6	47.3	44.6	47.3		43.4	44.7	47.1	44.6		40.1	55.2	46.9	46.5	
**States**															
Selangor	144	178	123	445	10.124^*^	101	100	45	246	6.624	79	43	47	169	12.878^*^
	32.3	33.6	35.7	33.7		29.2	27.4	26.5	27.9		27.3	21.4	32.0	26.5	
WPKL	79	118	85	282		72	61	43	176		59	32	36	127	
	17.7	22.3	24.6	21.4		20.8	16.7	25.3	20.0		20.4	15.9	24.5	19.9	
Perak	223	233	137	593		173	204	82	459		151	126	64	341	
	50.0	44.0	39.7	44.9		50.0	55.9	48.2	52.1		52.2	62.7	43.5	53.5	
**Gross household income**															
< RM 1,500	183	240	123	546	18.080	169	157	79	405	25.282^**^	149	104	65	318	11.713
	46.4	50.2	38.9	46.0		52.0	47.0	49.7	49.5		54.6	56.5	48.5	53.8	
RM 1,500–RM 3,000	120	122	102	344		96	96	48	240		77	42	42	161	
	30.5	25.5	32.3	29.0		29.5	28.7	30.2	29.3		28.2	22.8	31.3	27.2	
RM 3,000–RM 5,000	34	58	44	136		20	47	19	86		21	24	19	64	
	8.6	12.1	13.9	11.4		6.2	14.1	11.9	10.5		7.7	13.0	14.2	10.8	
More than RM 5,000	46	51	38	135		25	32	11	68		20	10	6	36	
	11.7	10.7	12.0	11.4		7.7	9.6	6.9	8.3		7.3	5.4	4.5	6.1	
**Highest education parent**															
Never schooled	1	2	0	3	10.106	2	0	0	2	19.728	0	1	0	1	10.453
	0.3	0.4	0	0.3		0.6	0	0	0.3		0.0	0.5	0.0	0.2	
Primary	23	28	15	66		25	17	5	47		15	14	5	34	
	6.1	6	4.9	5.7		8.1	5.2	3.2	5.9		5.7	7.7	3.9	5.9	
Lower secondary	83	83	58	224		77	58	33	168		50	44	28	122	
	22.1	17.8	19	19.5		24.9	17.8	21.2	21.22		18.9	24.2	21.7	21.2	
Upper secondary	168	210	128	506		134	156	77	367		136	80	61	277	
	44.7	45.1	41.8	44.1		43.4	47.9	49.4	46.4		51.5	44	47.3	48.2	
Pre-university	27	36	35	98		28	24	13	65		26	12	10	48	
	7.2	7.7	11.4	8.5		9.1	7.4	8.3	8.2		9.8	6.6	7.8	8.3	
Degree/Master/PhD	71	102	68	241		40	69	27	136		36	29	24	89	
	18.9	21.9	22.2	21		12.9	21.2	17.3	17.2		13.6	15.9	18.6	15.5	
Others	3	5	2	10		3	2	1	6		1	2	1	4	
	0.8	1.1	0.7	0.9		1	0.6	0.6	0.8		0.4	1.1	0.8	0.7	

*** p < 0.001,

** p < 0.01,

* *p < 0.05*.

The median (lower(Q1) and upper(Q3) quartile) values for each CVD risk factor are presented by clusters for each year in Table 2. Since the data deviated from normal/Gaussian distribution, the median differences between clusters were compared using the non-parametric Kruskal Wallis test. Each component of the CVD risk factors (blood pressure, body composition and blood lipid) differed by clusters. In each year, statistically significant differences (ρ < 0.001) were found between at least one pair of clusters in all three components of biological CVD risk factors. The high risk clusters for each year comprised of subjects with the highest median (Q1, Q3) for each CVD risk factor measured. The systolic blood pressure among students from high risk clusters were consistently higher across the years; 118.00 (110.00, 122.00) in 2012, 112.00 (108.00, 120.00) in 2014 and 114.00 (108.50, 122.00) in 2016. The median body fat and waist circumference values in all three study periods were distinctly higher among students of the high risk clusters (BF : 37.80 (31.00, 43.90) in 2012, 38.95 (30.80, 45.40) in 2014 and 33.00 (26.20, 41.00) in 2016; WC : 83.00 (75.00, 89.00) in 2012, 86.85 (80.00, 95.50) in 2014 and 81.50 (72.25, 89.75) in 2016. Median HDL for high risk clusters in all years are reported to be 1.30 (1.10, 1.41) in 2012, 1.20 (1.10, 1.40) in 2014, and 1.30 (1.10, 1.50) in 2016. In *post-hoc* multiple comparisons, the high risk clusters were found to be statistically significantly different from the low and medium risk clusters (*post-hoc* test, *p* < 0.05).

**Table 2:** Median differences in individual risk factors between clusters in 2012, 2014, and 2016, Kruskal Wallis (KW) and results of *post-hoc* multiple comparisons.^†^

	2012					2014					2016				
	Low *n* = 446	Medium *n* = 529	High *n* = 345	Total *n* = 1,320	χ^2^^*^	Low *n* = 346	Medium *n* = 365	High *n* = 170	Total *n* = 881	χ^2^^*^	Low *n* = 289	Medium *n* = 201	High *n* = 147	Total *n* = 637	χ^2^^*^
	Median (Q1, Q3)	Median (Q1, Q3)	Median (Q1, Q3)	Median (Q1, Q3)		Median (Q1, Q3)	Median (Q1, Q3)	Median (Q1, Q3)	Median (Q1, Q3)		Median (Q1, Q3)	Median (Q1, Q3)	Median (Q1, Q3)	Median (Q1, Q3)	
SBP	100.00^a^^,^ ^c^	110.00^a^^,^^b^	118.00^b^^,^^c^	110.00	489.143	96.00^a^^,^^c^	110.00^a^^,^^b^	112.00^b^^,^^c^	105.00	336.682	99.00^a^^,^^c^	115.00^a^	114.00^c^	108.00	320.770
	(94.00, 109.00)	(108.0, 120.0)	(110.00, 122.00)	(100.00, 118.00)		(90.00, 100.00)	(100.00, 120.00)	(108.00, 120.00)	(98.00, 114.00)		(93.00, 105.00)	(110.00, 121.00)	(108.50, 122.00)	(100.00, 117.00)	
DBP	60.00^a^^,^^c^	70.00^a^^,^^b^	72.00^b^^,^^c^	70.00	466.575	60.00^a^^,^^c^	70.00^a^^,^^b^	70.00^b^^,^^c^	66.00	322.221	60.00^a^^,^^c^	71.00^a^	70.00^c^	66.00	339.320
	(54.00, 65.00)	(65.00, 76.00)	(70.00, 80.00)	(60.00, 75.00)		(52.00, 60.00)	(63.00, 73.00)	(68.00, 78.00)	(60.00, 70.00)		(57.00, 64.00)	(69.00, 77.00)	(65.00, 78.00)	(60.00, 71.00)	
Body Fat	17.75^c^	17.10^b^	37.80^b^^,^^c^	21.35	583.435	21.75^a^^,^^c^	23.50^a^^,^^b^	38.95^b^^,^^c^	24.70	230.245	23.90^c^	21.30^b^	33.00^b^^,^^c^	24.80	116.338
	(12.40, 22.80)	(10.90, 23.90)	(31.00, 43.90)	(13.80, 30.75)		(15.00, 26.80)	(13.20, 30.10)	(30.80, 45.40)	(17.30, 32.10)		(19.10, 28.10)	(12.40, 28.00)	(26.20, 41.00)	(18.50, 31.10)	
Waist	62.50^c^	63.00^b^	83.00^b^^,^^c^	65.00	569.716	65.50^a^^,^^c^	68.00^a^^,^^b^	86.85^b^^,^^c^	69.00	329.069	65.50^a^^,^^c^	65.50^a^^,^^b^	81.50^b^^,^^c^	68.00	162.974
	(59.00, 67.00)	(59.50, 68.00)	(75.00, 89.00)	(60.10, 75.00)		(62.00, 65.50)	(63.80, 74.00)	(80.00, 95.50)	(63.70, 77.50)		(61.80, 70.00)	(61.00, 74.00)	(72.25, 89.75)	(62.50, 76.00)	
HDL	1.40^a^^,^^c^	1.60^a^^,^^b^	1.30^b^^,^^c^	1.45	330.500	1.40^a^^,^^c^	1.60^a^^,^^b^	1.20^b^^,^^c^	1.40	240.725	1.50^c^	1.50^b^	1.30^b^^,^^c^	1.50	84.624
	(1.24, 1.60)	(1.41, 1.80)	(1.10, 1.41)	(1.29, 1.70)		(1.20, 1.50)	(1.40, 1.80)	(1.10, 1.40)	(1.20, 1.60)		(1.40, 1.70)	(1.30, 1.70)	(1.10, 1.50)	(1.30, 1.70)	
LDL	2.87^a^^,^^c^	2.36^a^^,^^b^	2.99^b^^,^^c^	2.69	254.959	2.80^a^^,^^c^	2.40^a^^,^^b^	3.30^b^^,^^c^	2.70	176.864	2.60^a^^,^^c^	2.30^a^^,^^b^	3.30^b^^,^^c^	2.60	143.372
	(2.47, 3.34)	(2.00, 2.73)	(2.56, 3.47)	(2.24, 3.16)		(2.40, 3.30)	(2.10, 2.80)	(2.90, 3.80)	(2.30, 3.20)		(2.10, 3.00)	(2.00, 2.70)	(2.90, 3.90)	(2.10, 3.10)	
TG	0.83^a^^,^^c^	0.69^a^^,^^b^	1.07^b^^,^^c^	0.83	263.275	0.80^a^^,^^c^	0.70^a^^,^^b^	1.10^b^^,^^c^	0.80	170.861	0.70^c^	0.70^b^	1.10^b^^,^^c^	0.80	125.316
	(0.64, 1.07)	(0.56, 0.88)	(0.86, 1.41)	(0.63, 1.07)		(0.60, 1.10)	(0.60, 0.90)	(0.90, 1.60)	(0.60, 1.10)		(0.60, 0.90)	(0.50, 0.80)	(0.80, 1.40)	(0.60, 1.00)	
TC: HDL	3.32^a^^,^^c^	2.68^a^^,^^b^	3.75^b^^,^^c^	3.13	664.559	3.40^a^^,^^c^	2.80^a^^,^^b^	4.20^b^^,^^c^	3.20	479.234	2.90^c^	2.80^b^	3.90^b^^,^^c^	3.00	250.478
	(3.00, 3.67)	(2.41, 2.94)	(3.33, 4.23)	(2.71, 3.62)		(3.10, 3.80)	(2.50, 3.10)	(3.80, 4.70)	(2.80, 3.80)		(2.60, 3.20)	(2.50, 3.10)	(3.50, 4.50)	(2.60, 3.50)	

* All clusters are significantly different from each other, p < 0.001. Values with common superscript letters are significantly different from each cluster (p < 0.05) after using Dunnett T3 procedure for post-hoc multiple comparisons.

a p < 0.05 between Low and Medium.

b p < 0.05 between Medium and High.

c p < 0.05 between Low and High.

† *KW between clusters by boys and girls found all clusters to be significantly different from each other (results not shown)*.

**Figure 2:**
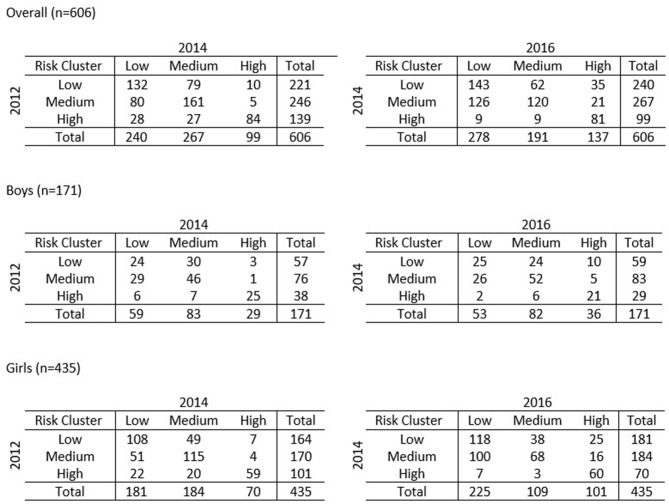
Tracking of students between clusters from 2012–2014 to 2014–2016.

Figure 2 shows the 3 × 3 matrices tracking the transitions of the students between 2012 and 2014 (13–15 years old) and 2014 to 2016 (15–17 years old). For the purpose of tracking, only students with complete data from 2012 to 2016 were considered. A total of 606 students, 171 boys and 435 girls, had complete data. In terms of locality, 276 (45.5%) students were from rural residential areas and 330 (54.5%) of them were from urban areas.

**Figure 3:**
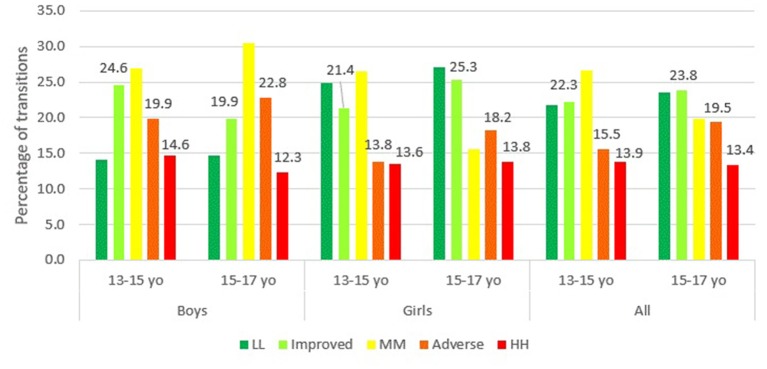
Transition of clusters from 2012–2014 (13–15 years old) to 2014–2016 (15–17 years old).

The transitions between risk clusters were categorized as either stagnant (or stable) (LL: low to low, MM: medium to medium and HH: high to high), improved (medium to low, high to low and high to medium) or adverse (low to medium, low to high, medium to high). Figure 3 shows the transitions between clusters from 2012 to 2014 and from 2014 to 2016. Overall, the number of subjects who remained at high risk was 13.9% from 2012 to 2014 and 13.4% from 2014 to 2016. The number of subjects who had adverse transitions increased from 15.5% (13–15 years) to 19.5% (15–17 years). For the girls, the number that remained at high risk over the two transition periods was about the same (13.6 vs. 13.8 %) whereas for boys, the percentage reduced from 14.6 to 12.3%. Adverse transitions among the girls increased quite a bit from 13.8% (2012–2014) to 18.2% (2014–2016). The same trend was observed among the boys (19.9%, 2012–2014 vs. 22.8%, 2014–2016). However, compared to girls, the transition among the boys to higher risk clusters were significantly greater. The improved transitions among the adolescents are also noteworthy. The number of those from the total sample who had improved transitions in the two transition periods were about the same (22.3%, 2012–2014 vs. 23.8%, 2014–2016). However, a marked decrease was observed among boys with 24.6% of them having improved transitions between the period 13–15 years old but only 19.9% between the period 15–17 years old. On the contrary, for girls, the number of those with improved transitions increased from the period between 13–15 and 15–17 years old (21.4 vs. 25.3 %).

Table 3 shows the percentage of change in cluster transitions by each biological CVD risk factor. Some large changes in percentage were observed between groups with improved and adverse transitions. The systolic and diastolic blood pressure for those in adverse transitions from 2012 to 2014 increased significantly from 10.00% (3.77,20.00) to 16.67% (3.45,16.67), respectively. On the contrary, the blood pressure levels for those in improved transitions reduced by 12.28% (−18.18, −6.57) for systolic and 16.67% (−25.36, −8.57) for diastolic. The median differences between all the transition groups were statistically significant (χ^2^ = 172.4, ρ < 0.001 *for systolic and χ*^2^ = 168.3, ρ < 0.001 *for diastolic*). In terms of body composition, only body fat showed significant differences in median values among the groups, χ^2^ = 46.2, ρ < 0.001 with median percentage change for the adverse transition of 13.03% (−3.35, 34.29). The median percentage change of triglyceride in adverse transitions was only 3.67% (−18.91, 25.00). As for transitions from 2014 to 2016, the Kruskal Wallis test showed statistically significant differences in percentage change in blood pressure (χ^2^ = 156.4, ρ < 0.001 *for systolic*, χ^2^ = 145.8, ρ < 0.001 *for diastolic*), body composition (χ^2^ = 37.2, ρ < 0.001 *for body fat*) and blood lipid (χ^2^ = 17.98, ρ < 0.001 *for HDL*). The median for the percent change were much higher in adverse transitions with 16.49% (5.00, 24.44) for systolic, 15.00% (1.67, 27.50) for diastolic and 4.43% (−6.43, 15.02) for body fat. As for percentage change in body lipid, the median in improved transitions was greatest for triglyceride at −11.11% (−25.00, 16.67).

**Table 3:** Median percentage of change in biological risk factors in groups with different transition patterns.

Percentage of change (%)—biological risk factors	2012–2014							2014–2016						
	LL	IMP	MM	ADV	HH	Total	χ^2^	LL	IMP	MM	ADV	HH	Total	χ^2^
	(*n* = 132)	(*n* = 135)	(*n* = 161)	(*n* = 94)	(*n* = 84)	(*n* = 606)		(*n* = 143)	(*n* = 144)	(*n* = 120)	(*n* = 118)	(*n* = 81)	(*n* = 606)	
	Median	Median	Median	Median	Median	Median		Median	Median	Median	Median	Median	Median	
	(Q1, Q3)	(Q1, Q3)	(Q1, Q3)	(Q1, Q3)	(Q1, Q3)	(Q1, Q3)		(Q1, Q3)	(Q1, Q3)	(Q1, Q3)	(Q1, Q3)	(Q1, Q3)	(Q1, Q3)	
Gender: Boys/ Girls, *n* (%)	24/108 (18.2/81.8)	42/93 (31.3/69.9)	46/115 (28.6/71.4)	34/60 (36.2/63.8)	25/59 (29.8/70.2)	171/435 (28.2/71.8)	10.165^†^	25/118 (17.5/82.5)	34/110 (23.6/76.4)	52/68 (43.3/56.7)	39/79 (33.1/56.7)	21/60 (25.9/74.1)	171/435 (28.2/71.8)	24.752^***^
SBP	−4.00	−12.28	0.00	10.00	0.00	−2.72	172.430^***^	1.90	−6.12	−0.83	16.49	0.00	1.00	156.385^***^
	(−10.00, 2.02)	(−18.18, −6.57)	(−9.09, 5.56)	(3.77, 20.00)	(−7.93, 8.33)	(−10.48, 7.95)		(−2.06, 9.69)	(−13.33, 2.00)	(−5.09, 9.09)	(5.00, 24.44)	(−6.15, 8.13)	(−6.06, 10.00)	
DBP	0.00	−16.67	0.00	16.67	0.00	0.00	168.363^***^	0.00	−8.96	1.34	15.00	−1.25	0.00	145.809^***^
	(−13.56, 8.53)	(−25.36, −8.57)	(−12.50, 11.11)	(3.45, 33.33)	(−12.50, 12.70)	(−14.29, 12.90)		(−6.67, 11.11)	(−18.66, 0.00)	(−5.71, 16.19)	(1.67, 27.50)	(−12.16, 8.97)	(−9.09, 14.29)	
Body Fat	21.54	7.14	15.45	13.03	1.52	11.39	46.240^***^	0.00	−2.88	0.37	4.43	−7.29	−0.59	37.168^***^
	(6.73, 40.98)	(−10.92, 25.35)	(−0.33, 33.80)	(−3.35, 34.29)	(−7.55, 10.12)	(−3.35, 32.58)		(−7.15, 8.45)	(−11.57, 6.87)	(−8.41, 8.96)	(−6.43, 15.02)	(−16.32, 0.22)	(−9.86, 8.33)	
Waist	5.26	3.89	5.17	4.45	5.69	4.72	7.556	−0.34	−0.82	−1.88	0.85	0.63	−0.34	7.749
	(−0.92, 10.31)	(−2.22, 8.79)	(0.31, 12.07)	(−0.88, 10.26)	(−0.46, 13.17)	(−0.77, 10.88)		(−4.95, 4.43)	(−6.66, 3.97)	(−6.97, 4.04)	(−5.07, 6.94)	(−5.52, 4.14)	(−6.07, 4.67)	
HDL	0.00	−1.52	−0.90	0.42	0.00	0.00	8.619^*^	6.25	−2.17	0.00	0.00	0.00	0.00	17.985^***^
	(−11.76, 8.33)	(−14.13, 7.14)	(−10.53, 7.53)	(−9.09, 15.38)	(−11.44, 7.69)	(−11.76, 8.33)		(0.00, 16.67)	(−9.76, 7.14)	(−12.13, 8.33)	(−7.69, 14.29)	(−7.14, 15.38)	(−25.00, 20.00)	
LDL	2.94	0.95	4.17	0.79	3.79	2.64	8.920^*^	−5.56	−6.25	−8.33	−3.18	0.00	−5.33	8.724^*^
	(−7.72, 14.01)	(−12.44, 13.15)	(−8.60, 16.50)	(−16.67, 8.47)	(−5.38, 15.99)	(−9.70, 13.64)		(−16.95, 4.45)	(−15.38, 5.41)	(−16.67, 4.17)	(−13.33, 7.69)	(−12.00, 7.14)	(−15.38, 5.56)	
TG	−0.38	−1.79	1.69	3.67	0.93	0.46	2.088	−8.33	−11.11	−11.11	0.00	0.00	0.00	6.629
	(−24.27, 27.02)	(−26.20, 31.05)	(−21.05, 36.84)	(−18.91, 25.00)	(−13.40, 29.99)	(−21.57, 31.58)		(−25.00, 12.50)	(−25.00, 16.67)	(−23.61, 20.00)	(−25.00, 37.50)	(−20.00, 15.38)	(−25.00, 20.00)	
TC : HDL	1.19	4.76	3.31	−2.57	5.46	2.28	14.691^**^	−9.38	−4.00	−3.57	−3.96	−4.44	−4.71	12.634^**^
	(−6.66, 12.26)	(−7.30, 14.44)	(−4.17, 11.11)	(−12.47, 9.51)	(−3.76, 16.29)	(−7.30, 12.97)		(−16.67, 0.00)	(−12.70, 4.76)	(−12.12, 3.18)	(−13.89, 6.45)	(−13.51, 2.44)	(−13.79, 3.85)	

††Percentage of change in risk factors deviated from normal distribution, presented as median(IQR) for each transition group, compared by non-parametric Kruskal Wallis H-test (Chi-square).

*** p < 0.001,

** p < 0.01,

* p < 0.10,

† *p < 0.05*.

## Discussion

This study investigated the clustering of biological CVD risk factors in a young population of adolescents in Malaysia, a country suffering from the heavy burden of mortality from CVD for more than 40 years (33). Clustering was defined according to eight biological CVD risk factors: systolic blood pressure, diastolic blood pressure, body fat, waist circumference, HDL cholesterol, LDL cholesterol, triglyceride and TC:HDL ratio. Many studies included only single risk factors such as blood pressure (17, 34), blood lipids (17, 35, 36), body composition (37–39), fasting blood glucose (35) or physical fitness (16, 40, 41) but only a few considered combinations of risk factors to form clustered cardiovascular risks (15–19, 42). The combination of risk factors that form meaningful clustering differ from one population to another and is chosen based on theoretical grounds for maximal discrimination between clusters (13, 27). Although metabolic syndrome is a form of clustering (19, 42), the pre-identified risk factors do not necessarily contribute to the clustering of the sample studied. For example, in this study, fasting blood glucose, a pre-requisite for metabolic syndrome, was found to contribute the least (13, 43) in explaining the differences between high, medium and low risk clusters for each screening year and was therefore omitted in clustering. This was perhaps due to low predictive values of the risk factor among the younger population.

The results of the present study suggest that clustering of biological CVD risk factors occur among adolescents and can be segmented into three risk clusters; low, medium and high. The young and relatively healthy population of adolescents aged 13–17 years old account for the comparatively small number of subjects in high risk clusters (*n* = 345 in 2012, *n* = 170 in 2014, and *n* = 147 in 2016). Nevertheless, it is important to take note that the standardized mean values of the final cluster centers of the high risk clusters were distinctively higher (*Z* = 0.618 in 2012, *Z* = 0.730 in 2014, and *Z* = 0.665 in 2016) compared to the mean cluster centers of the low and medium risk clusters. Furthermore, the *post-hoc* multiple comparison test of differences concurred with statistically significant differences between the high-risk clusters and other lower risk clusters. Thus, we can conclude that students belonging to high risk clusters are more susceptible to attaining cardiovascular diseases during adulthood.

Once the clusters were identified, it was interesting to track the number of subjects who maintained their cluster membership or moved toward higher or lower ranks over the 5-years (2012–2016) tracking period. The adolescent cohort showed stability among younger boys but noticeable transitions among older boys. Contrarily, substantial drifts in cluster transitions observed among younger girls turned out to be slightly better among older girls. The different patterns of tracking among boys and girls probably adhere to the different physiological growth between genders. These findings concur with a 10-years study of trends of risk factors among school children in Serbia (44) that found the proportion of waist circumference increase to be higher among younger girls compared to older girls. As for boys, a later take-off in growth spurt cause rampant transitions only later in adolescent period. However, in principal, the overall tracking pattern between clusters in both transition periods was quite low. Basically, this tells us that both boys and girls in this population do not remain in their ranks of risk cluster but keep changing ranks over time. The possible explanation could be that in addition to maturation and physiological development, lifestyle changes especially in physical activity and dietary intake among boys and girls vary erratically causing instability in rank orders of the clusters.

On top of tracking the movements or transitions of clusters, it was even more important to categorize and monitor these transitions as either stagnant (stable), adverse or improved. The findings from this longitudinal observation of the transitions are intriguing. Notably, the number of Malaysian adolescents who remained at high risk clusters and had adverse transitions over the 5-years cluster tracking period was reasonably high especially in the risk factors for body composition. The proportion of adolescents from the MyHeARTs study who maintained in high risk cluster ranks over 5 years were about 13.5–14%. The Cardiovascular Risk in Young Finns study (17) found 25% of adolescents remaining in high risk tertiles over 6 years whereas the Bogalusa Heart study (18) found about 61% of them staying in high risk quartiles over 8 years. Although a smaller proportion of Malaysian adolescents remained in high risk cluster over time as compared to adolescents from Finland and United States, these results may not be comparable due to different clustering methodologies and combinations of risk factors.

In the present study, as the adolescents grew older, the percentage of those in adverse transitions increased from 15.5 to 19.5%. Particularly among the teenage boys, the percentage increased from 19.9 to 22.8% and among the teenage girls, it increased from 13.8 to 18.2%. These group of adolescents may be many folds at higher risk of CVD in the future. As such, it is essential to monitor the developmental record of these adolescents until young adulthood. The observed trend among these school-going adolescents could be due to various factors accrued during their teenage age. Among the few are sedentary lifestyle that includes lack of exercise, hours of passive television watching, video gaming and long digital hours involving gadgets and social media (45). Depending on personal preferences, the level of physical inactivity for each adolescent differ by gender. This perhaps explain the higher number of adverse transitions among boys compared to girls. Apart from that, unhealthy dietary intake and imbalanced nutrition contribute vastly to high risk and adverse transitions. In general, adolescent diet has been reported to be high in fat and sugar and low in vitamins and minerals (23, 46). Large quantities of fast food consumption and energy dense food is a reason for high-calorie diet in most adolescents (47). A systematic review on Malaysian adolescents revealed that high consumption of energy, macronutrients and skipping meals have an effect on cardio-metabolic health (48). Overall, the interaction of sedentary lifestyle and unhealthy nutrition are deemed to be major influences in the increasing prevalence of risk profiles among the younger generation (49). Regular health screenings should be conducted in schools to monitor adolescents at potential risk of poor health. Also, reward programmes could be implemented for health promoting and nutrient-friendly schools. Parents play an equally important role in inculcating healthy eating habits and promoting an active lifestyle among children.

### Strengths and Limitations

This study is one of the first to investigate the longitudinal clustering of CVD risk factors among adolescents in Malaysia. The selection of relevant risk factors and the clustering method of the present study makes it unique and have some important features in comparison to other studies that investigated clustering of biological CVD risk factors. Most studies clustered individuals based on different criteria, such as cumulative risk scoring by percentile or cut-off points (9, 50, 51). These methods have limitations that incur loss of information due to restricted threshold values (9, 51). Even if a child falls behind by merely 1 unit measurement of a high risk factor, the child falls out of the high-risk group. This somewhat reduces capturing the actual number of children closest to the higher extreme end of the distribution. The hierarchical and k-means clustering is not based on dichotomization of high risk factors and simple additive risk scoring. Instead, both steps allow synergistical interaction of risk factors in multiple combinations to classify subjects into different risk clusters. K-means clustering splits the combined information of the risk factors into risk clusters where all data points in each cluster are closest to its' cluster center that relates to the mean of the cluster (52). The combined methods merge the two most similar observations with the smallest increase in overall within-cluster variance and simultaneously maximizes between-cluster variance (25, 27). These clustering methods serve as an alternative to risk factor clustering (13). To the best of our knowledge, none of the past studies have taken the approach of clustering a single individual with multiple risk factors of CVD as done in this study. This makes this study unique. The hierarchical and non-hierarchical clustering method empower other clustering techniques and are found to be more robust in terms of segregating adolescents into high, medium and low risk groups (27). The strength of this study lies in the method used and overcomes the limitation of categorization of risk factors that leads to loss of power and reduced stability (9). Since the proportion of Malay students were more than 75% in each screening year, results from this study may not mirror the racial composition of Malaysian adolescents. The low representation of the Chinese and Indian adolescents can be seen as a limitation to this study.

## Conclusion

Reliable and meaningful clustering of multiple risk factors of CVD is of practical importance especially since it allows us to identify adolescents at high risk of CVD. These adolescents who remain in high risk profiles and move to higher risk clusters over time are at greater risk of developing cardiovascular diseases in adulthood. Findings from the study is hoped to be useful in forming relevant strategies to reduce the rate of cardiovascular diseases in adulthood by starting preventive measures during childhood. The perception that the young do not need medical attention is something of the past and should be corrected to be given equal care.

## Data Availability Statement

The dataset analyzed for the current study is not publicly available due to participants confidentiality and privacy. Requests to access the datasets should be directed to the corresponding author.

## Ethics Statement

The study was approved by the Ethics Committee, University of Malaya Medical Centre (ethical approval number-14-376-20486).

## Consent For Publication

All participants, along with their parents, provided oral assent, and written consent prior to participating.

## Author Contributions

HM, TS, NA-S, and MJ designed the study. HM and MJ collected the data. NT and KC conducted the analysis. NT, KC, and HM interpreted the data. NT wrote the manuscript. All authors read and approved the final manuscript.

### Conflict of Interest

The authors declare that the research was conducted in the absence of any commercial or financial relationships that could be construed as a potential conflict of interest.
